# Investigation of N-polar InGaN growth on misoriented ScAlMgO_4_ substrates

**DOI:** 10.1038/s41598-023-46542-w

**Published:** 2023-11-07

**Authors:** Mohammed A. Najmi, Pavel Kirilenko, Daisuke Iida, Kazuhiro Ohkawa

**Affiliations:** Electrical and Computer Engineering Program, Computer, Electrical and Mathematical Sciences and Engineering Division, King Abdullah University of Science and Technology (KAUST), 23955-6900 Thuwal, Saudi Arabia

**Keywords:** Materials for optics, Lasers, LEDs and light sources, Inorganic LEDs, Semiconductor lasers

## Abstract

We report the growth of N-polar InGaN layers on misoriented ScAlMgO_4_ (SAM) substrates with offset of 0.3 to 5.8° toward the *m*-plane. The surface of N-polar InGaN with small-offset substrates exhibited hexagonal hillocks similar to those commonly observed in N-polar GaN layers. Larger misorientation angles resulted in smoother surfaces of the InGaN layers. In contrast, the crystalline quality of InGaN indicated an opposite trend with significantly improved quality observed at smaller misorientation angles. We obtained an unprecedented crystalline quality of N-polar InGaN using SAM substrates with a 0.5° offset, which exhibited a ${000}\overline{2}$ X-ray rocking curve full width at half maximum value of 223 arcsec. The crystalline quality and surface morphology of InGaN were significantly influenced by the step surface of substrates according to atomic force microscopy observations.

## Introduction

ScAlMgO_4_ (SAM) substrates are promising candidates for high-quality InGaN layers, which have great potential for high InN-molar-fraction InGaN-based yellow–red light-emitting diodes (LEDs) and laser diodes (LDs)^1^. In general, high InN-molar-fraction in InGaN active regions results in a larger in-plane lattice constant, which introduces significant defects due to increased lattice mismatch with the underlying GaN layers. This poses a significant challenge in the development of InGaN-based LEDs and LDs with longer wavelengths.

Underlying layers of strain-relaxed InGaN can improve the quality of InGaN quantum wells (QWs) and In incorporation, which is very attractive for growing yellow–red InGaN-based light emitters^1–5^. One approach to overcome the challenge of strain is to use the emerging SAM substrates. These substrates have exhibited structural, optical, and thermal properties that make them compatible with growing lattice-matched with In_0.17_Ga_0.83_N^1,6^. Polarity control is also important, especially for emitters with longer wavelength^7–12^. It is possible to increase In incorporation in the InGaN crystal by growing an N-polar InGaN layer instead of a Ga-polar layer^7,10,11,13^. N-polar LEDs have demonstrated low-forward-voltage operation and a low droop effect compared to Ga-polar LEDs^14,15^.

The quality of InGaN on SAM substrates is still not sufficient for optoelectronic devices^1,6,16^. The full width at half maximum (FWHM) in X-ray rocking curves (XRC) of InGaN (0002) has typically reached high values ranging from 2500 to 3000 arcsec^6,16^. Previously, we demonstrated N-polar InGaN grown on a SAM substrate without any buffer layers, which exhibited low FWHM values of XRCs when using a sliced substrate surface^17^. Low FWHMs of ${000}\overline{2}$ and ${10}\overline{1}\overline{2}$ XRCs of 384 arcsec and 481 arcsec were obtained, respectively^17^. However, the N-polar InGaN layers still suffer from a high density of hexagonal hillocks, which make the surface rough^17,18^. This issue leads to variation in the depth of the quantum wells, resulting in different InN-molar-fraction caused by phase separation, which could be a detrimental issue for light emitters. Considerable efforts have been reported for InGaN growth on SAM substrates with the aim of growth optimization to realize high crystalline quality and smooth surface morphology.

In this work, N-polar InGaN layers were grown on misoriented SAM substrates with different offset angles. The surface morphology was investigated to study the influence of the substrate offset on the surface roughness, and InGaN layers with smooth surfaces were obtained using SAM substrate with high misorientation. To understand the mechanism behind the improvement in surface morphology, we investigated the initial growth of InGaN on SAM substrates. InGaN nucleation and the surface morphology of the SAM substrates with different offsets revealed the behavior of the InGaN growth. Both ${000}\overline{2}$ symmetric and ${10}\overline{1}\overline{2}$ asymmetric XRC FWHM values were used to quantitatively compare the crystalline quality of InGaN layers, which indicated superior quality for an InGaN layer grown on a SAM substrate with a small offset. Transmission electron microscopy (TEM) analysis was also used to examine the epitaxial interface and InGaN polarity and explain the microscopical characteristics of the crystalline quality that was identified by XRC and atomic force microscopy (AFM).

## Results and discussion

**Figure 1:**
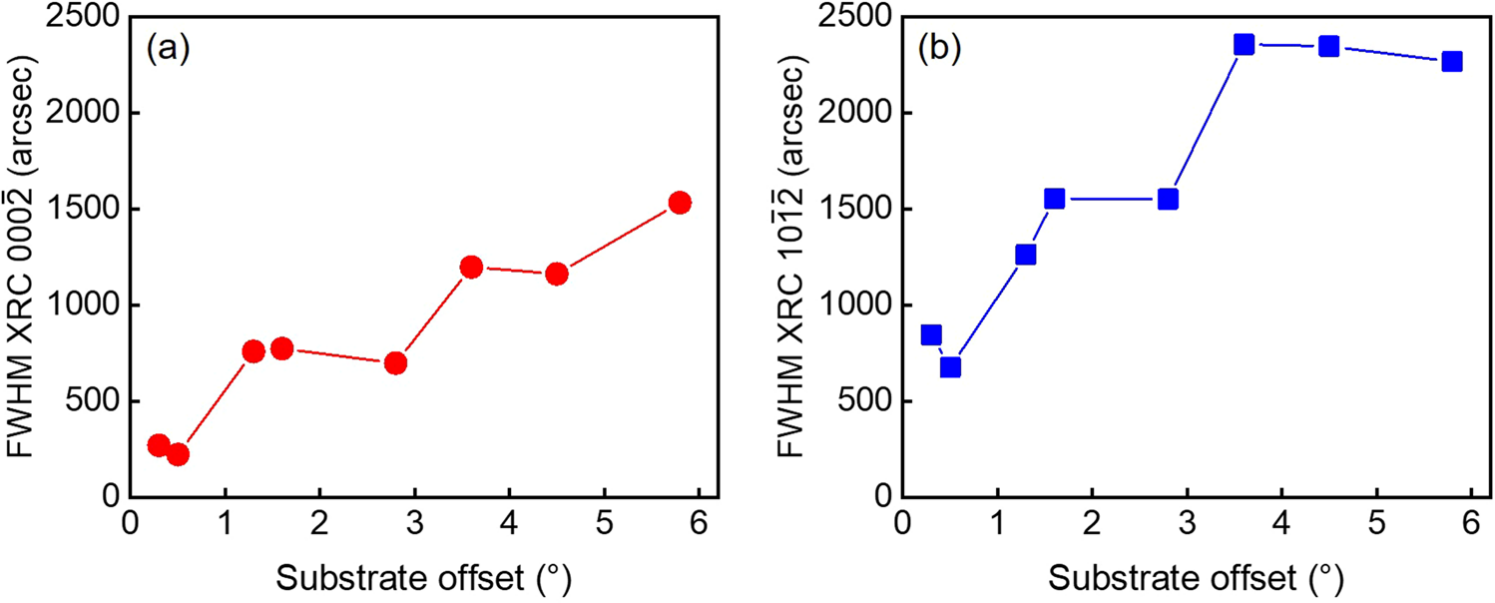
(**a**) Symmetric ${000}\overline{2}$ and (**b**) asymmetric ${10}\overline{1}\overline{2}$ XRC FWHM values of InGaN on SAM substrates with different misorientation angles.

**Figure 2:**
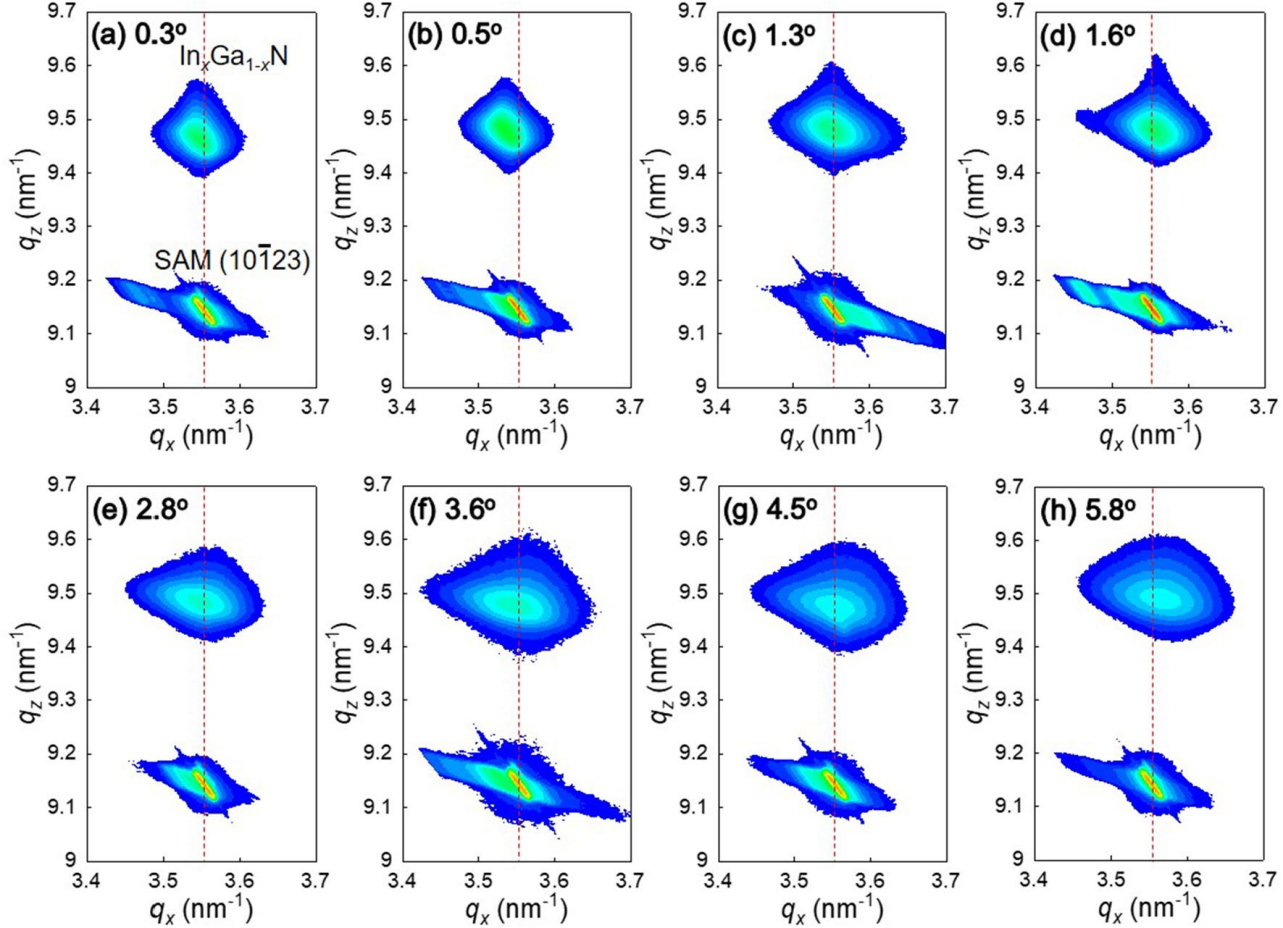
${10}\overline{1}\overline{5}$ reflection X-ray reciprocal space maps of InGaN layers on SAM substrates with different misorientation angles. The red dashed lines indicate the in-plane SAM lattice constant. The lattice parameters of the *a*- and *c*-axes are calculated by $\frac{2}{\sqrt{3}{q}_{x}}$ and $\frac{5}{{q}_{z}}$, respectively.

Figure 1 shows the FWHM of the symmetric ${000}\overline{2}$ and asymmetric ${10}\overline{1}\overline{2}$ XRCs that were used to evaluate the crystalline quality of InGaN layers grown on misoriented SAM substrates with offset angles ranging from 0.3 to 5.8°. Both symmetric and asymmetric XRCs exhibited similar trends of an increase in the FWHMs as the SAM offsets increased. Notably, high crystalline quality of InGaN layers was obtained when they were grown on SAM with an offset of 0.5°. The ${000}\overline{2}$ and ${10}\overline{1}\overline{2}$ XRC FWHMs indicate the crystalline quality of InGaN on SAM with values of 223 arcsec and 677 arcsec, respectively. These small XRC FWHM values demonstrate a significant improvement in the crystalline quality of InGaN and superior InGaN growth on the SAM substrate. The ${000}\overline{2}$ XRC FWHM value is comparable to that of typical Ga-polar GaN on sapphire^19–21^. The X-ray reciprocal space map represents the *a*- and *c*-lattice parameters, as shown in Fig. 2. In the InGaN on SAM, the strain is significantly reduced compared with the typical InGaN/GaN system. Here, it is noted that the InGaN layers on SAM should be unstrained when the InN-molar-fraction in InGaN is 0.17 which corresponds to the in-plane lattice matching. We found that the strain in InGaN layers (with InN-molar-fractions ranging from 0.15 to 0.19) on SAM was small, ranging from 1.8 × 10^–4^ to 2.8 × 10^–3^. For instance, the strain in In_0.17_Ga_0.83_N on GaN structures is 1.9 × 10^–2^. Therefore, we consider that all of the InGaN layers exhibited nearly coherent growth on the SAM substrates.

**Figure 3:**
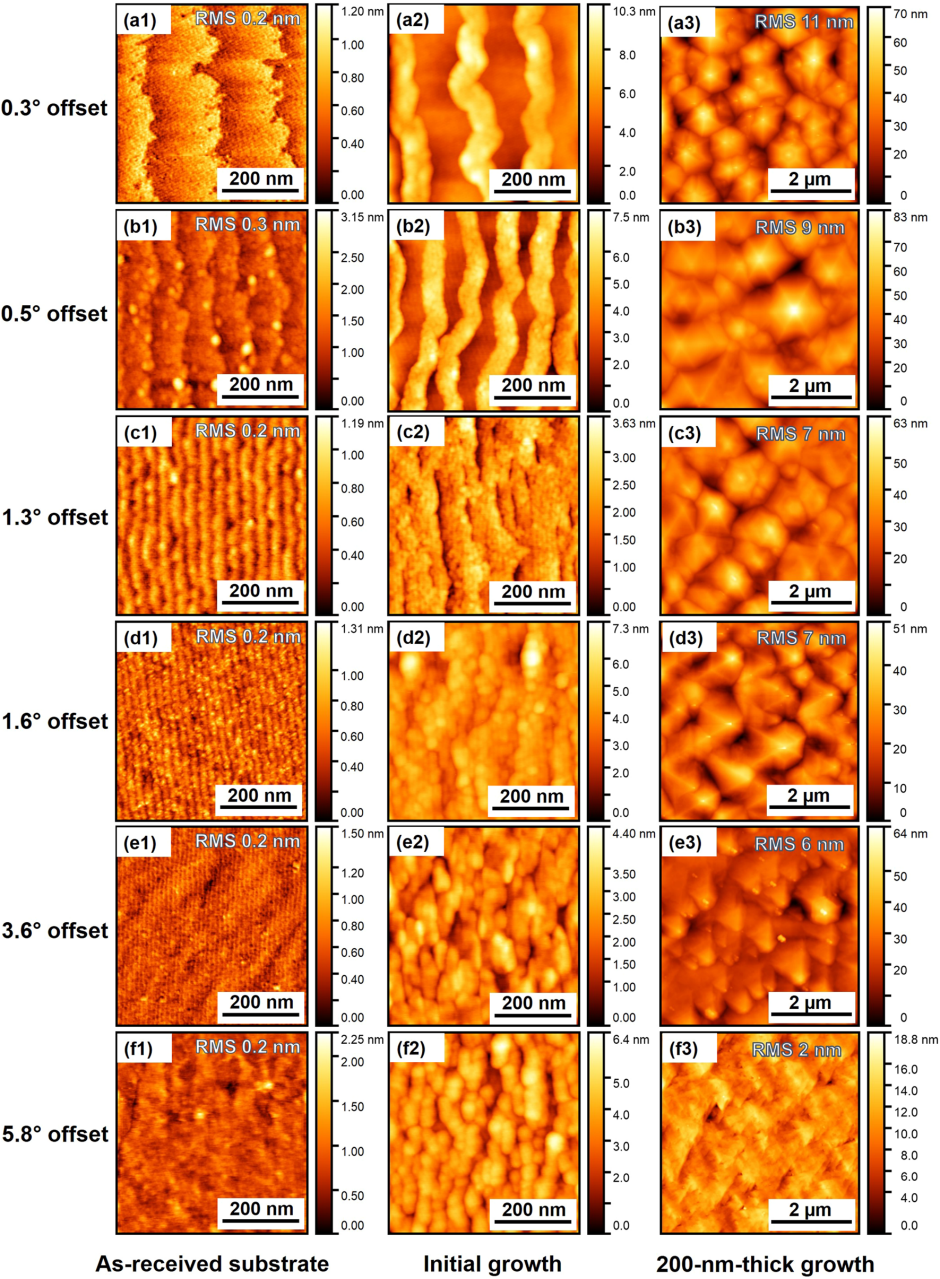
AFM images of (a1-f1) as-received substrate, (a2-f2) 10 s of InGaN growth, and (a3-f3) 20 min of InGaN growth with different misorientation angles.

Despite the improvement in crystalline quality with smaller SAM misorientation, the InGaN on SAM with a 0.3° offset had a rougher surface than the one obtained with a 5.8° offset, as shown in Fig. 3. The substrate misorientation on the surface morphologies of SAM substrates and InGaN layers were characterized using AFM. In addition, AFM was used to trace the development in time of InGaN growth with the different substrate offsets. The initial growth can clearly be traced back to the origin of the morphological distinction observed by AFM and revealed divergence among the InGaN layers grown on SAM with different offsets.

We found that the initial growth modes of InGaN were significantly influenced by the terrace width of substrates. The terrace widths of the substrates narrowed as the offset angles increased from 0.3 to 3.6°, resulting in approximate shifts from 176 to 13 nm, respectively, as shown in Fig. 3a1–e1. However, in the case of the 5.8° offset, it was very difficult to identify the width. We obtained the step height and terrace width of the 5.8° offset substrate via high-angle annular dark-field scanning transmission electron microscopy (HAADF-STEM) observation (Supplementary information, Fig. S1). The step height and terrace width were approximately 0.8 nm and 7.4 nm, respectively. It is found that the step height corresponds to the 1/3 of the *c*-lattice constant of the substrate. These terraces play a significant role in epitaxial growth, particularly during the initial stages of growth, which start at the step edges.

In general, InGaN growth requires the growth temperature to be decreased to incorporate In atoms into the crystals. Our N-polar InGaN growth was also performed with a low flow rate of NH_3_ to enhance the surface migration of atoms and reduce zinc-blende phase^22^. The surface migration of atoms is an important factor in determining the material quality. A high NH_3_ supply increases N species but also increases the concentration of hydrogen in the growth atmosphere, which means that In incorporation into InGaN could be reduced^13^.

Figure 3a2,b2 show that the initial InGaN was grown along with step edges of SAM. For low misorientation angle substrates, the lined three-dimensional InGaN nuclei lead to bending of the dislocations toward the crystal's sidewall surfaces, effectively terminating their progression to the surfaces, and thereby improving the crystalline quality as shown by narrow XRC results. However, this dislocation bending process is only allowed along with the *m*-axis direction. Also, the growth of high-density tiny islands occurred when the substrate offset exceeded 1.6°. Smaller terrace width facilitated the conjoining of InGaN nuclei on the terrace surfaces, leading to the presence of numerous grain boundaries. Thus, the XRC FWHM values became larger when the substrate offset angles increased. We found that the crystalline quality of InGaN layers on SAM substrates is significantly affected in the initial growth of the InGaN layers.

The root mean square (RMS) values of the surface roughness decreased from 11 to 2 nm when InGaN layers were grown on SAM substrates with offset ranging from 0.3 to 5.8°. This phenomenon is similar to that of N-polar GaN growth on misoriented sapphire substrates with up to 4° of offset^23,24^. This surface improvement is highly advantageous for the growth of InGaN QWs, which require a flat surface with a reduced height of hexagonal hillocks, typical for N-polar InGaN. As these results show, N-polar InGaN growth on misoriented SAM substrates exhibits a trade-off between crystallinity and surface smoothness. This phenomenon is also demonstrated in N-polar GaN on misoriented sapphire substrates^24^.

**Figure 4:**
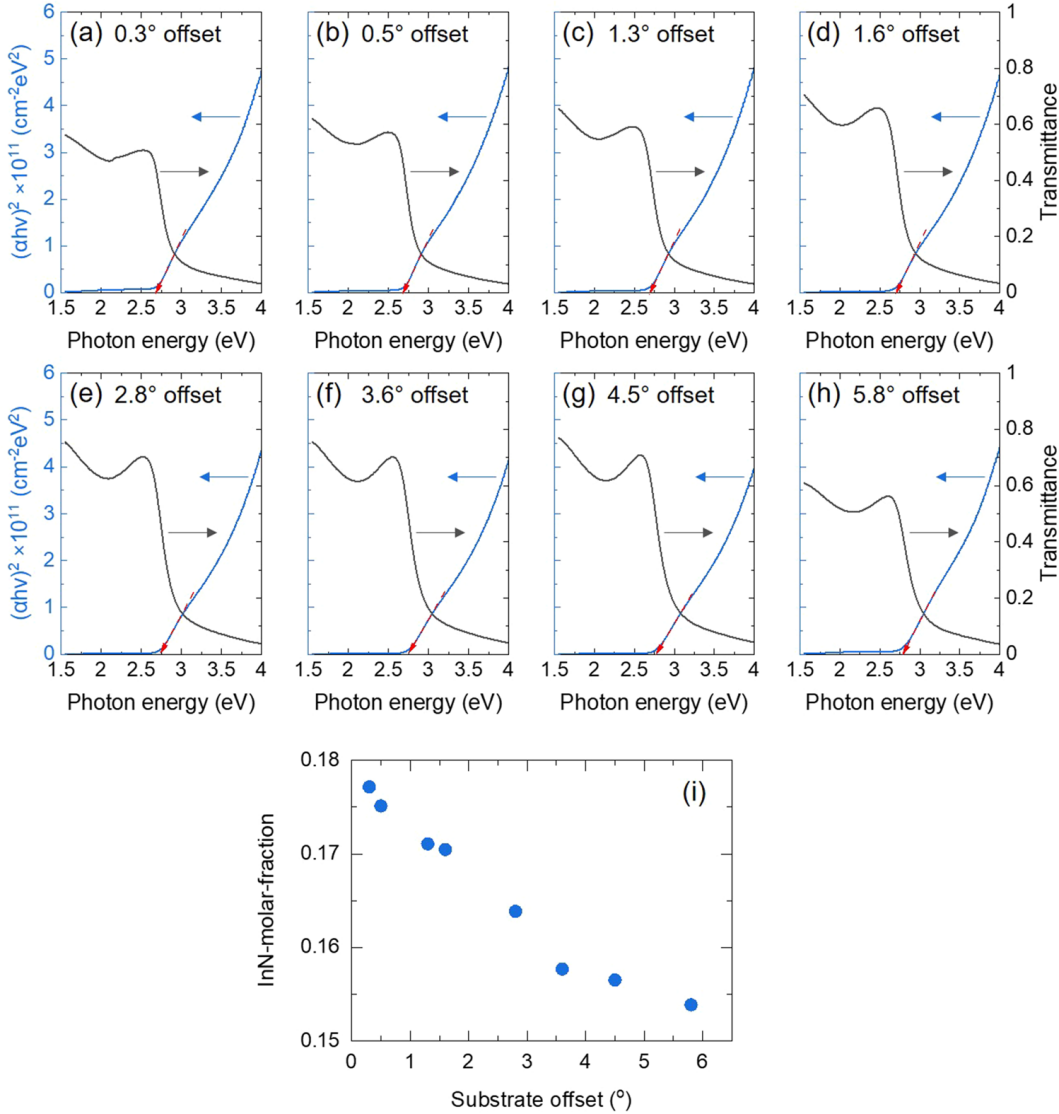
(**a**-**h**) Transmittance and Tauc plots for the determination of InGaN bandgap. (**i**) InN-molar-fraction in InGaN layers with different misorientation angles.

Figure 4 shows the optical transmittance properties of InGaN layers with different substrate offsets. The transmittance is able to determine the material band gap energy. Tauc plot is the conventional method for the estimation of material bandgap from the optical properties such as transmittance, reflectance, and absorption given by spectrophotometer^25,26^. We calculated Tauc plot using Eq. (1) and (2).

1 $$T=A\cdot exp(-\alpha d)$$

2 $${(\alpha h\nu )}^{1/n}=C\cdot (h\nu -{E}_{g})$$

where *T* is the transmittance, *A* is a constant, α is the absorption coefficient, *d* is material thickness, h is the Planck constant, ν is photon frequency, *C* is a constant, and *E*_*g*_ is bandgap energy. The *n* was adopted 1/2 because InGaN is a direct bandgap material^27^. The InGaN bandgap energy was derived from each Tauc plot, then we calculated the InN-molar-fraction using Eq. (3).

3 $${E}_{g,InGaN}={xE}_{g,InN}+{(1-x)E}_{g,GaN}-bx(1-x)$$

where *E*_*g*,InGaN_ is the bandgap energy of the In_*x*_Ga_1-*x*_N, *E*_*g*,InN_ is the bandgap energy of the InN, *E*_*g*,GaN_ is the bandgap energy of the GaN, *x* is the InN-molar-fraction in In_*x*_Ga_1-*x*_N, and *b* is the bandgap bowing parameter of In_*x*_Ga_1-*x*_N. The bandgap bowing parameter b was adopted as 1.7^28^. Figure 4i indicates the InN molar-fraction in InGaN layers with the different substrate offsets. We found that the InN-molar-fraction in InGaN was shifted to be lower with the substrate offsets increase. The result suggests that the Ga and In metal atoms are exposed to the InGaN step-edge surfaces, especially, In atoms desorbed due to the high volatility. It means that the InN-molar fraction in InGaN should be reduced as the density of the step-edge increases. The tendency of this result is the same as the typical Ga-polar InGaN on misoriented GaN substrates^29,30^. In addition, the growth rate of InGaN layers on 0.3° offset substrate was approximately 10% greater than that of InGaN on 5.8° offset substrate as observed via STEM. Therefore, the reduction of the InN-molar-fraction in InGaN grown on SAM substrates with a high offset angle (5.8°) is due to the lower growth rate with the step-edges increase.

**Figure 5:**
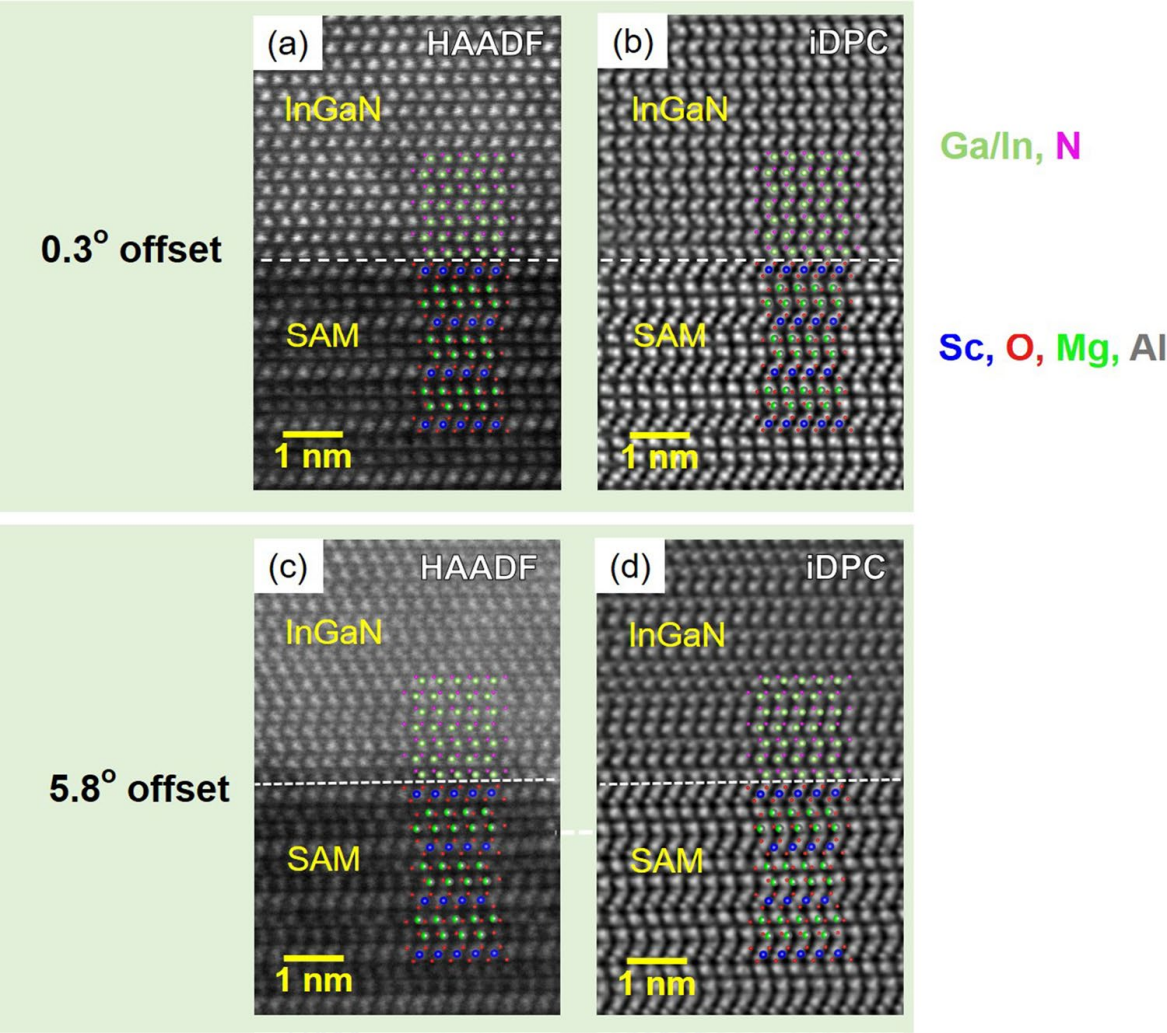
InGaN/SAM interface observation and InGaN polarity confirmation using (**a**, **c**) HAADF-STEM and (**b**, **d**) iDPC-STEM images of InGaN on SAM substrates (0.3° and 5.8° offsets). The interface is marked by a white dashed line.

To obtain InGaN layer polarity, a STEM analysis was performed with integrated differential phase contrast (iDPC). As shown in Fig. 5, the InGaN layer grown on the SAM with 0.3° offset was observed at the interface and showed InGaN polarity. In the HAADF-STEM image, contrast is generated by the atomic weight of the elements, and iDPC-STEM was used to identify the lighter elements. The polarity of the InGaN layer was identified as N-polar by the iDPC, which is identical to our previous work^18^. The SAM substrate is terminated with Sc-O bonds, and the metal (In, Ga) from the InGaN layer is epitaxially bound to the oxygen of the SAM substrate.

**Figure 6:**
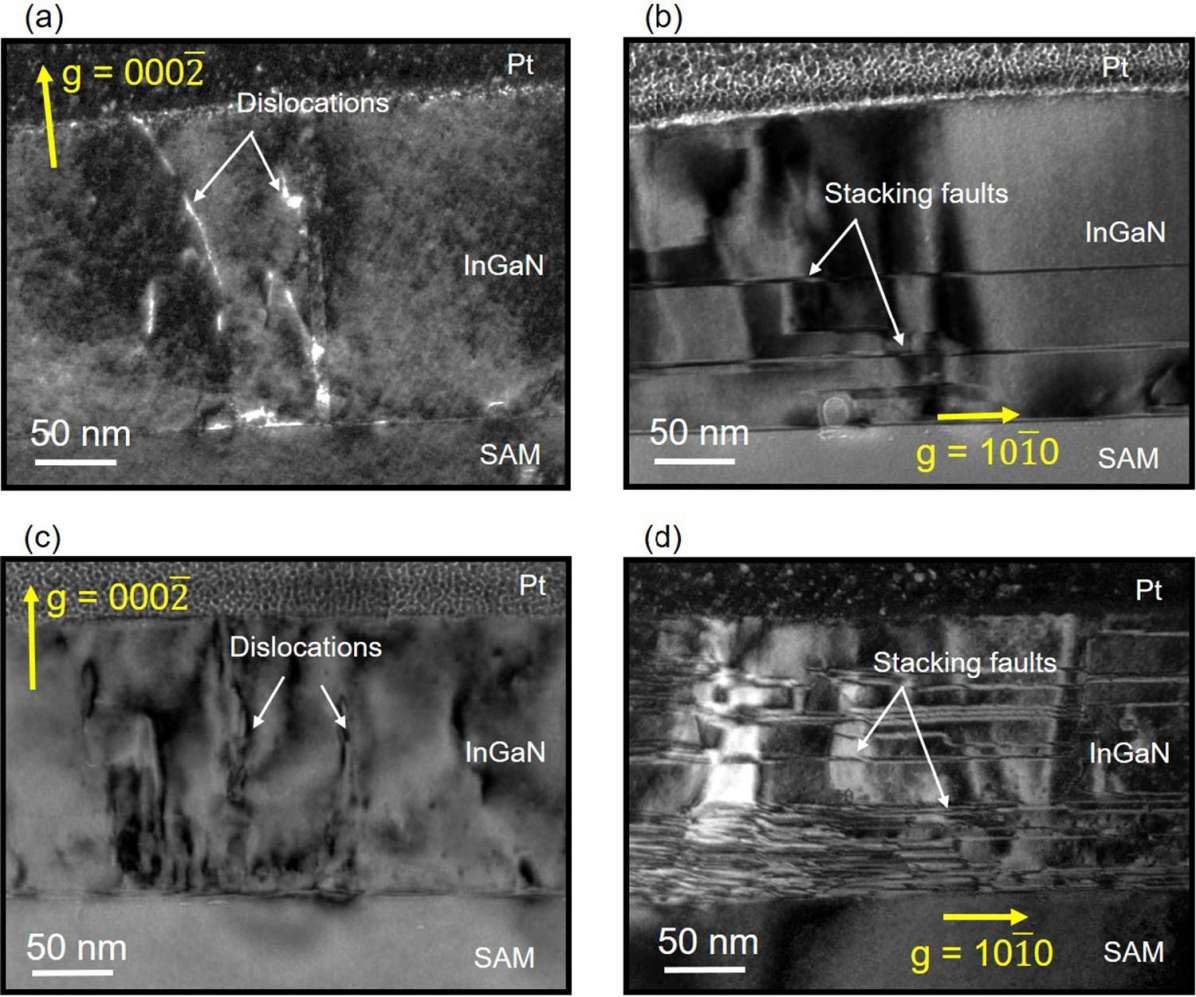
Dark-field cross-sectional TEM images with (**a**, **c**) g = ${000}\overline{2}$ and (**b**, **d**) g = ${10}\overline{1}0$ of InGaN on SAM substrates with 0.3° and 5.8° offsets, respectively.

We investigated the deterioration in crystalline quality that occurs when using misoriented SAM substrates. Dark-field TEM observations were used to analyze InGaN layers on SAM with 0.3° and 5.8° offsets, as shown in Fig. 6. Microstructural analysis can provide additional information to complement the XRC results. Figure 6a shows that the InGaN layer on SAM with the 0.3° offset is obviously distinctive and has less defects, which indicates high crystallinity. In contrast, the InGaN layer grown on SAM with 5.8° misorientation has inferior crystalline quality. The threading dislocations are increased, as shown in Fig. 6c, and this crystalline deterioration is in a good agreement with the XRC results. The microscopy observations are consistent with the deterioration of crystalline quality of the InGaN on the SAM with 5.8° misorientation revealed by the greater FWHM of the XRC.

In addition, the InGaN layer and SAM substrate with 0.3° misorientation presented a few stacking faults after the initial growth stage, whereas the InGaN on SAM with 5.8° offset included a high density of stacking faults near the InGaN/SAM interface. This observation suggests that the tiny domains can easily invert during the initial coalescence process. Also, we suggest other possibilities that the formation of the basal-plane stacking faults was caused by In segregation due to low surface migration of atoms due to a low growth temperature^31,32^. According to the AFM observations, we believe that the low surface migration of the atoms on substrates with 5.8° offset was due to the small terraces’ width. Also, N-polar growth potentially enhanced the In-incorporation rate compared with Ga-polar growth, which suggests that In segregation could occur easily in N-polar InGaN.

## Conclusions

We investigated the influence of SAM substrates with misorientation for N-polar InGaN growth. The surface morphologies of InGaN were significantly improved by increasing the substrates’ offset angles. InGaN layers exhibited different surface morphology characteristics in the initial growth stage, which revealed variation of the growth modes due to differences in terrace widths. The crystalline quality of N-polar InGaN obtained with 0.5° offset exhibited a ${000}\overline{2}$ X-ray rocking curve FWHM value of 223 arcsec. However, the crystallinity of InGaN degraded with increasing substrate misorientation angles up to 5.8°.

We observed basal stacking faults in InGaN on SAM with an offset angle of 5.8° due to lower surface migration. The results showed a trade-off between crystalline quality and surface roughness. The ultimate goal is to achieve both a flat surface and high crystallinity, which requires further optimization of the growth conditions, especially in regard to the surface migration of atoms.

## Methods

### Epitaxial growth of InGaN layers

All of the InGaN layers were epitaxially grown simultaneously on SAM substrates (Fukuda Crystal Laboratory) with different offset angles in the same metalorganic vapor-phase epitaxy reactor. The InGaN layers were grown on SAM substrates without any buffer layers in order to obtain N-polarity. The SAM substrates were prepared with misorientation offset angles of 0.3°, 0.5°, 1.3°, 1.6°, 2.8°, 3.6°, 4.5°, and 5.8° toward the *m*-axis. The precursors for Ga, In, and N were trimethylgallium (TMGa), trimethylindium (TMIn), and NH_3_, respectively.

Prior to growing the InGaN layers, the SAM substrates were thermally cleaned at 1000 °C for 5 min in H_2_. The atmosphere was then changed to N_2_, and the temperature was decreased to 830 °C. Once the temperature stabilized, NH_3_ gas was fed for 2 min for surface nitridation, and then the InGaN layers were grown on the substrates. The V/III and TMIn/(TMGa + TMIn) vapor ratios during the InGaN growth were 2000 and 0.63, respectively.

### Material characterization

The surface morphologies of SAM substrates, initial growth of InGaN, and 200-nm-thick InGaN were characterized by AFM using a Bruker Dimension Icon SPM. The transmittance of InGaN layers were analyzed by Ultraviolet–visible spectroscopy (UV-2600i, Shimadzu). The InN-molar-fraction of InGaN layers were determined by Tauc plot^25,26^. The crystalline quality of InGaN was measured by a Bruker D8 Discover X-ray Diffractometer equipped with a Göbel mirror and Ge (022) monochromator with Cu K_α1_ radiation (λ = 1.5418 Å). 2*θ*-*ω* scans were used to identify the InGaN peaks and substrate peaks for calibration and to obtain a reference angle of diffraction to compare the compositional variation among InGaN layers. Symmetric and asymmetric XRCs were used to quantitatively compare the crystalline quality based on the FWHMs. Reciprocal space mappings in ${10}\overline{1}\overline{5}$ reflection were used to observe the strain behavior.

### Electron microscopy characterization

For TEM investigation, a cross-section lamella was prepared with a focused ion beam scanning electron microscope (FEI, Helios G4) equipped with a field emission gun. HAADF-STEM images were obtained by double aberration-corrected TEM (Thermo Fisher Scientific, Titan Themis Z). The interfacial structure and polarity of the InGaN/SAM sample were identified by iDPC-STEM. TEM analysis was carried out at an operating voltage of 300 kV.

### Supplementary Information

Supplementary Information.

## Data Availability

The data that support the findings of this study are available from the corresponding author upon reasonable request.
