# Supplementary material for: Investigation of N-polar InGaN growth on misoriented ScAlMgO4 substrates
Source: Sci Rep. 2023 Nov 7;13:19332. doi: 10.1038/s41598-023-46542-w (PMC10630384; doi:10.1038/s41598-023-46542-w)
Supplement: Supplementary file 1 — Supplementary Information. [file 41598_2023_46542_MOESM1_ESM.docx]

**(Supplementary Information)**

**Investigation of N-polar InGaN growth on misoriented ScAlMgO_4_ substrates**

**Mohammed A. Najmi, Pavel Kirilenko, Daisuke Iida, and Kazuhiro Ohkawa^*^**

Electrical and Computer Engineering Program, Computer, Electrical and Mathematical Sciences and Engineering Division, King Abdullah University of Science and Technology (KAUST), Thuwal 23955-6900, Saudi Arabia

*kazuhiro.ohkawa@kaust.edu.sa

The step height and terrace width for the ScAlMgO_4_ (SAM) with 5.8° offsets were identified by high-angle annular dark-field scanning transmission electron microscopy (HAADF-STEM) observation, as shown in Fig. S1. The result clearly indicates the lattices of the materials, the top surface of the SAM substrate is terminated with Sc-O bonds. We found that the step height and terrace width were approximately 0.8 nm and 7.4 nm, respectively. It is noted that the step height corresponded to the 1/3 of the *c*-lattice constant of the SAM.


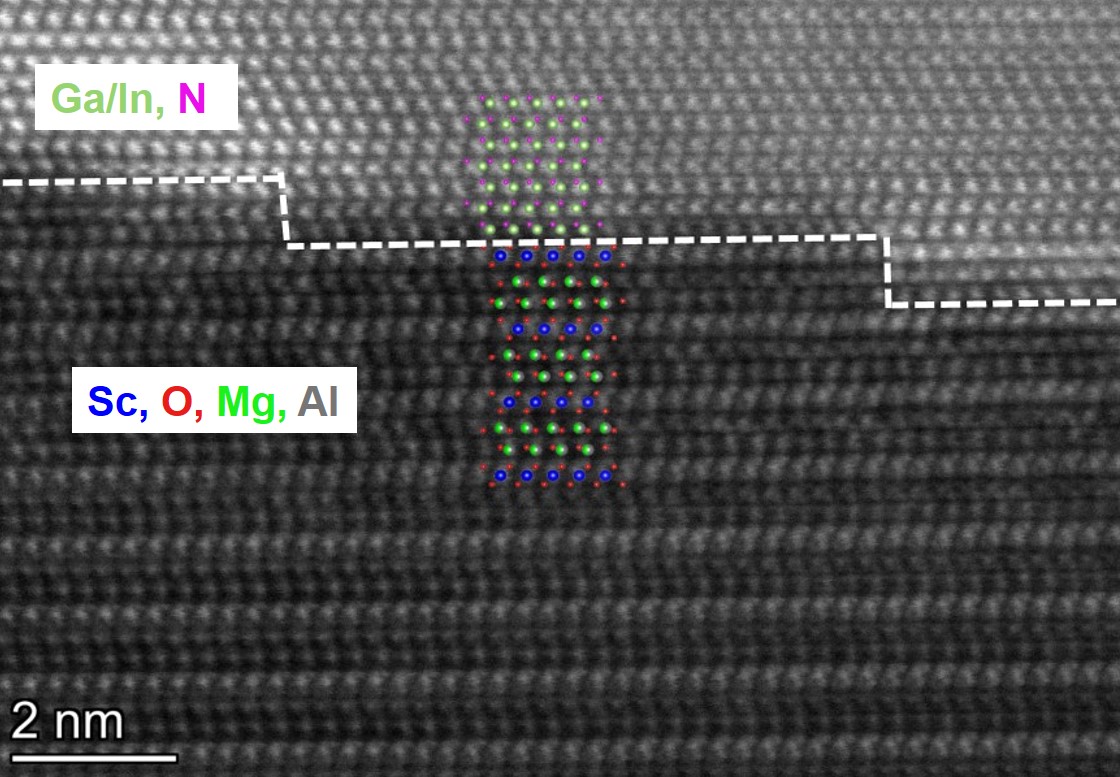


**Figure S1.** InGaN/SAM (5.8° offsets) interface observation using HAADF-STEM. The interface is marked by a white dashed line.
